# The barriers and facilitators of implementing a national laboratory-based AMR surveillance system in Cambodia: key informants’ perspectives and assessments of microbiology laboratories

**DOI:** 10.3389/fpubh.2023.1332423

**Published:** 2023-12-21

**Authors:** Sovathiro Mao, Chansovannara Soputhy, Sokreaksa Lay, Jan Jacobs, Grace Marie Ku, Darapheak Chau, Chhorvann Chhea, Por Ir

**Affiliations:** ^1^National Institute of Public Health, Phnom Penh, Cambodia; ^2^Institute of Tropical Medicine Antwerp, Antwerp, Belgium; ^3^Department of Microbiology, Immunology, and Transplantation, KU Leuven, Leuven, Belgium; ^4^Department of Frailty in Ageing Research, Vrije Universiteit Brussel, Brussels, Belgium

**Keywords:** antimicrobial resistance, laboratory-based surveillance, qualitative research, laboratory capacity assessments, clinical microbiology

## Abstract

**Background:**

Collecting data on antimicrobial resistance (AMR) is an essential approach for defining the scope of the AMR problem, developing evidence-based interventions and detecting new and emerging resistances. Our study aimed to identify key factors influencing the implementation of a laboratory-based AMR surveillance system in Cambodia. This will add additional insights to the development of a sustainable and effective national AMR surveillance system in Cambodia and other low- and middle-income countries.

**Methods:**

Key informants with a role in governing or contributing data to the laboratory-based surveillance system were interviewed. Emerging themes were identified using the framework analysis method. Laboratories contributing to the AMR surveillance system were assessed on their capacity to conduct quality testing and report data. The laboratory assessment tool (LAT), developed by the World Health Organisation (WHO), was adapted for assessment of a diagnostic microbiology laboratory covering quality management, financial and human resources, data management, microbiology testing performance and surveillance capacity.

**Results:**

Key informants identified inadequate access to laboratory supplies, an unsustainable financing system, limited capacity to collect representative data and a weak workforce to be the main barriers to implementing an effective surveillance system. Consistent engagement between microbiology staff and clinicians were reported to be a key factor in generating more representative data for the surveillance system. The laboratory assessments identified issues with quality assurance and data analysis which may reduce the quality of data being sent to the surveillance system and limit the facility-level utilisation of aggregated data. A weak surveillance network and poor guidance for outbreak response were also identified, which can reduce the laboratories’ opportunities in detecting critical or emerging resistance occurring in the community or outside of the hospital’s geographical coverage.

**Conclusion:**

This study identified two primary concerns: ensuring a sustainable and quality functioning of microbiology services at public healthcare facilities and overcoming sampling bias at sentinel sites. These issues hinder Cambodia’s national AMR surveillance system from generating reliable evidence to incorporate into public health measures or clinical interventions. These findings suggest that more investments need to be made into microbiology diagnostics and to reform current surveillance strategies for enhanced sampling of AMR cases at hospitals.

## Introduction

Antimicrobial resistance (AMR) is one of the leading global health threats; in 2019, an estimated 4.95 million deaths were associated with drug-resistant infections (1). The burden of AMR is disproportionately higher in low- and middle-income countries (LMICs) due to flagrant drug consumptions and utilisations (2). A study analysing global antibiotic consumption demonstrated that consumption in LMICs more than doubled from 2000 to 2015, reaching a rate of 24.5 billion defined daily doses (3). In Southeast Asia, the increase in AMR has also been attributed to socio-economic development and unregulated access to antimicrobials (4). Cambodia, in particular, has a high potential for emerging AMR threats due to its high prevalence of infectious diseases. A Cambodian paediatric hospital retrospectively analysed bacterial infections over a 9-year period and found that *Acinetobacter baumannii* had a resistance ratio of 93.3% towards third-generation cephalosporins while 62.1% of *Klebsiella pneumoniae* isolates were resistant to both ampicillin and gentamicin (5). Furthermore, a study from 2017 investigating animal products at Banteay Meanchey, a rural province in Cambodia, found that 52% of *Salmonella* isolated from these samples were multidrug-resistant (6). Although data on AMR is still scarce, available evidence from Cambodia and neighbouring countries suggests that the burden of AMR in Cambodia is likely to be high (7).

**Figure 1:**
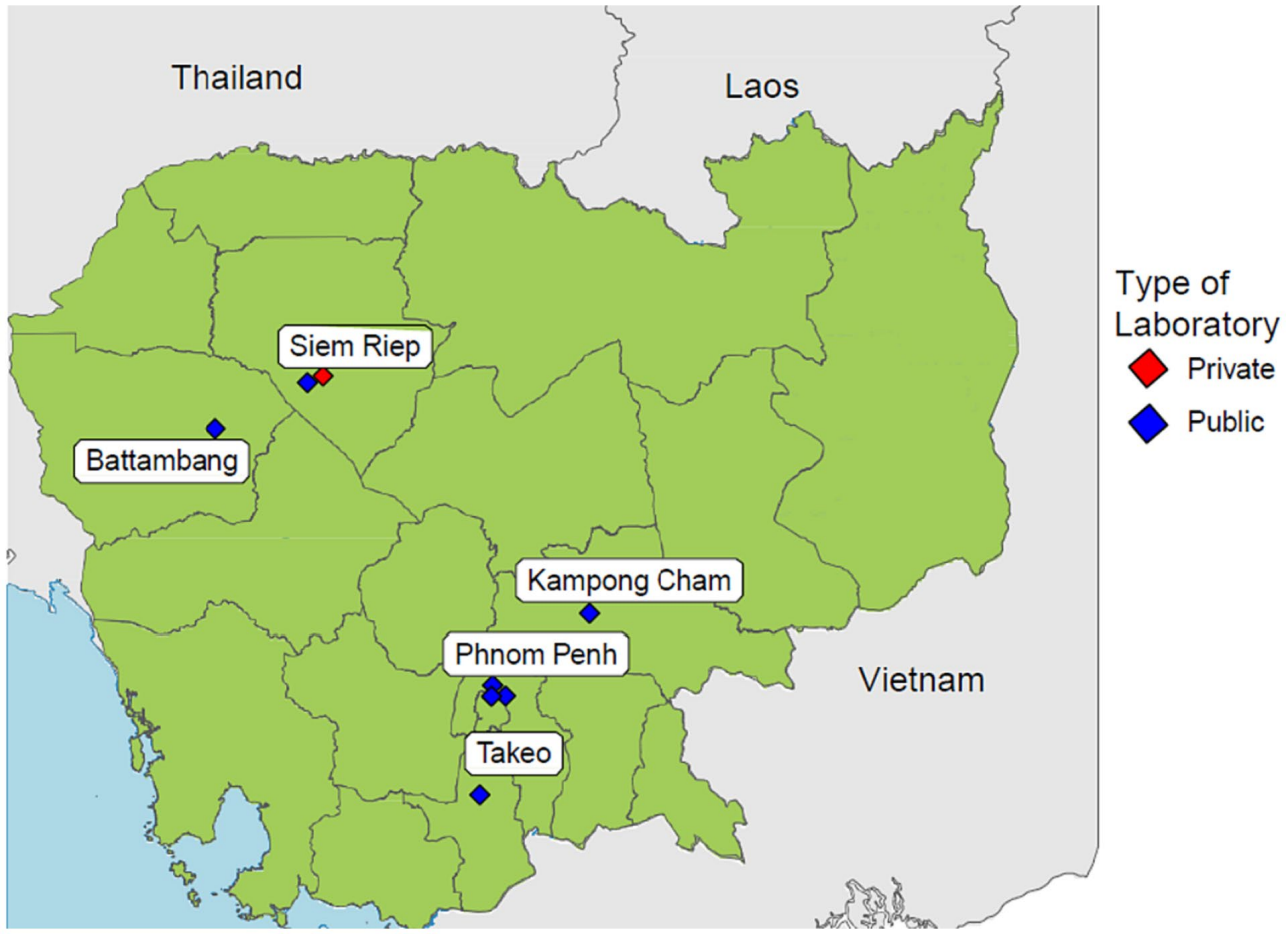
Distribution of all 8 sentinel sites participating in Cambodia’s national AMR surveillance system.

Cambodia has made great efforts in combating AMR by developing and implementing the Multi-Sectoral Action Plan on Antimicrobial Resistance and establishing a national AMR surveillance system (8). This national AMR surveillance system was fully established in 2018 by the Communicable Disease Control Department of the Ministry of Health. The surveillance system is a laboratory-based surveillance consisting of 8 sentinel sites distributed across the country, while the Communicable Disease Control Department serves as the coordinating centre responsible for governance and data aggregations (9). The geographical distribution of each sentinel site is shown in Figure 1. Cases are identified routinely when laboratory tests are positive for any of the 7 priority pathogens. The system collects microbiology laboratory data at all 8 sentinel sites while inpatient admission and specimen request ratios are collected from 4 sentinel sites. The National Reference Laboratory (NRL) is also a component of the surveillance system which enhances quality assurance by conducting confirmatory tests for isolates sent by sentinel sites. The national AMR surveillance system in Cambodia has successfully continued its operations likely due to contributing factors such as stakeholder accountabilities fostered by numerous policies and guidelines, financial and technical assistance from international partners and strong commitment to combat AMR by the government (10). Ultimately, this surveillance system aims to measure the health burden of AMR, provide data to inform interventions, and detect emerging resistance.

Conducting routine AMR surveillance at the national level enables the country to characterise the geographical and temporal patterns and magnitude of AMR. This information is essential for the development and refinement of public health policies and programs aimed at reducing AMR as well as guiding local empirical antimicrobial therapies (11, 12). However, implementing a robust AMR surveillance system capable of generating high quality data is challenging especially for LMICs due to weak microbiology laboratory capacity and governance, and sub-optimal availability of resources and investments (13, 14). A review of AMR research from Cambodia in 2019 suggested that unstandardised data, absence of data on important resistance mechanisms and low sample numbers will be an obstacle for utilising available data to inform local strategies (7). Additionally, the distribution of Cambodia’s sentinel sites further limits the system in generating nationwide-representative results, as they are situated only in urban areas or large provinces (10). These are some of the challenges that need to be overcome in order to develop a surveillance system capable of accurately measuring the scope of the AMR burden in Cambodia and effectively guiding interventions. However, to the best of our knowledge, there are no existing studies which explores the perspectives of those who are directly involved with managing, operating or using a laboratory-based AMR surveillance system in Cambodia. This study intends to fill this important gap in literature by incorporating these perspectives with quantitative assessments. It aims to determine the barriers and facilitators to implementing a laboratory-based AMR surveillance system in Cambodia to support the development of an effective AMR surveillance system.

## Methods

### Study design and settings

We applied concurrent mixed qualitative-quantitative methods to have a more nuanced perspective of barriers and facilitators influencing development of an effective AMR surveillance system in Cambodia. The qualitative aspect of the study included document review and analysis, direct field observations and key informant interviews (KII). For document review and analysis, policy documents such as standard operating procedures (SOP) guidelines, national action plans and reports related to Cambodia’s AMR surveillance system were reviewed using qualitative document analysis pre- and post- interviews. This was done to gain insights into the key themes and objectives of the national AMR surveillance system in Cambodia, gather more information during interviews and identify any discrepancies between guidelines and the associated implementation. Semi-structured interviews with key informants (KI) were conducted to gain a deep understanding of the perspectives of national level individuals involved with policy development and implementers of the National AMR surveillance system. Field observations at sentinel sites and the Communicable Disease Control Department also gave detailed insights into the reporting and management of AMR data such as the use of paper-based records, digital platforms to store the AMR data and how these two are integrated at the central level. Observations were recorded and stored in field notes. Together, this data triangulation provided the source of data for qualitative analysis on the factors influencing AMR surveillance system implementation for this study. In addition, we further assessed the AMR surveillance system by evaluating the capacity of microbiology laboratories using an assessment tool. This allows an in-depth analysis of the various technical issues faced by laboratories participating in the surveillance system. Permission for conducting the assessment was given by and conducted at 6 sentinel sites.

### Participant selection and characteristics

As a first step, stakeholder analysis was conducted by reviewing the Multi-Sectoral Action Plan on Antimicrobial Resistance in Cambodia 2019–2023 and the SOP for Cambodia Laboratory-based Surveillance resulting in a total number of 25 potential informants (9). KI were purposively selected on the following criteria: a current role in the national AMR surveillance system or work involving antibiotic resistance and the utilisations of data collected by the surveillance system. To gain comprehensive understanding of the challenges of implementation, it was essential that we investigate all aspects of the surveillance system’s structure from sample collection to data analysis and data utilisation. As such, prospective respondents included a diverse group of laboratory staff, individuals who interact with and manage the AMR surveillance database, hospital management with influence over the surveillance implementation, potential users of AMR surveillance data, and those who work at the central level to govern and monitor the progress of the surveillance system. We classified our study population into two groups: Surveillance personnel (SP) and high-level management (HM). SPs were directly involved with contributing to the collection of surveillance data and routine functioning of the system. HMs were individuals entitled to make governing decisions and influence policies, designs or resource allocations related to the national AMR surveillance system. KI were contacted by an invitation letter as well as by a phone call for confirmation. 22 of the 25 identified KI accepted the invitation and allowed the interviews. The three KI which did not accept the invitation, due to conflicting schedules, provided contacts of replacements who had similar roles and backgrounds. Invitations for interviews were terminated when data saturation was reached, and no additional themes could be identified. All 25 KIIwere conducted between October 2022 and February 2023.

### Data collection

#### Key informant interviews

Two semi-structured interview guides asking on similar elements but tailored for SPs and HMs were developed. The interview guides consisted of open-ended questions categorised into four different sections: (1) Role and organisational involvement in AMR surveillance in Cambodia. (2) Surveillance resources. (3) Surveillance function and data management. (4) Perceptions and recommendations for AMR surveillance in Cambodia. Guides for HMs includes an additional fifth section with questions on the application and utilisations of surveillance data. The semi-structured interview format was used to allow for a degree of flexibility in exploring themes due to the diverse professional background of respondents. Each guide was developed in English and translated to Khmer (Supplementary files).

To improve appropriateness of the interview guides, they were piloted with two microbiology staff: the head of microbiology unit at the national reference lab and a laboratory technician whose role involves data entry and reporting of AMR trends. Their characteristics matches well with some of the study population.

Key informant interviews took place in each interviewee’s office and through video calls on Zoom when in-person interviews are not possible. All interviews were conducted in the respondents’ native language (Khmer or English) and took between 45 and 60 min. Each interview was conducted by two researchers – an interviewer (SM) and a note taker (CS or SL). All three authors have had prior training and experience in qualitative analysis. SM is a male researcher at the NIPH and has no established relationship or connections to the AMR surveillance system or individuals working within it. CS is a male researcher and a surveillance officer for the influenza-like illnesses (ILI) and severe acute respiratory infection (SARI) surveillance system. Their experience with the ILI and SARI surveillance system may result in pre-conceived notions of the factors that influence the AMR surveillance systems. Both SM and CS considers themselves as outsiders to the AMR surveillance system. SL is a female quality officer at the NRL and has established relationships with some key informants identified in the study. SL may be biased towards her experience as a staff at the NRL as well as having worked with certain key informants in the past. Their experience of working within the Cambodian health system may also confer assumption of resource constraints and their effects. All three were encouraged to conduct the study without any assumptions about the surveillance system prior to the start of the interviews. To reduce the influence of individuals’ positionality, more than one researcher conducted the KII and qualitative analysis. Furthermore, interviewers used probes sparingly and in a neutral and respectful manner to allow the informants to express their own views when expanding on their ideas.

Interviews were recorded, manually transcribed, and translated verbatim by SC and SM. Interviews which did not receive consent to be audio recorded were written in an interview note format in Khmer and also translated into English. SM proofread the translated transcripts. There were no repeat or follow-up interviews during the period of this study. To reduce misinterpretations, notes made during the interview were debriefed and further discussed among the research team post-interview. KI were not given the option to read transcripts before analysis. Instead, respondent validations were conducted after qualitative analysis to increase validity of our interpretations. Preliminary themes were summarised and shown to respondents by an email or a text message containing a link to an online survey (QualtricsXM, Provo, UT). KIs were asked to identify errors or gaps in interpretations and provide additional information on the challenges of implementing AMR surveillance which have not been identified in the initial analysis.

#### Laboratory assessment

We developed our assessment tool by adapting the WHO’s Laboratory Assessment Tool (LAT) for facilities to reflect laboratory functions which are relevant to the needs of the AMR surveillance system as set out by surveillance system’s SOP (15). The adapted LAT contains 10 different modules with a total of 293 questions. These are closed questions which can be answered with 1. Yes, 2. Partial, 3. No or 4. Not Applicable (NA). Some indicators had responses of 1, 2 and 3. Responses with Yes or 1 were given a score of 100%, 2 were given a score of 50% while 3 or No were given a score of 0%. NA responses were not included in the calculations. The laboratory quality management system team and microbiology unit of the NRL were consulted to support the tool’s development.

Laboratory assessments were conducted by an assessment team comprised of four individuals with experience in laboratory quality management and clinical microbiology. The assessments took two working days to complete for each sentinel site. Field observations, document inspections and short interviews were used to collect data for the assessment tool. Prior to each assessment, laboratories were requested to nominate one laboratory technician and one quality officer who could answer questions on day-to-day operations. Each interview took between thirty minutes and one hour and questions were directly from the LAT. Overall, 6 interviews were conducted with 12 laboratory staffs for the assessment.

#### Data management and analysis

A framework analysis of the key informant interview data was conducted starting with data familiarisation through multiple re-readings of the interview transcripts. To accurately report the situation, the primary and secondary author conducted field visits alongside the laboratory assessment team to fully immerse in the situation of the surveillance system. We utilised a data-driven method by performing an initial round of inductive coding on three transcripts resulting in codes which are specific to the AMR surveillance system in Cambodia. This formed the initial analytical framework. Deductive coding of more transcripts was performed, making sure to note any passages or codes which do not fit into the existing set of codes. This process of inductive and deductive coding was iterated until no new codes were generated resulting in the final analytical framework. This analytical framework was applied to the transcripts and charted into a framework matrix enabling cross-case analyses and developments of memos. Memos and codes in the framework matrix were built into themes which were revisited and refined consistently throughout the process (Table 1). Framework analysis was led by SM and SC using Microsoft Word and Microsoft Excel. Results from validation survey were considered as additional data and contributed to the refinements of emerging themes. Following the completion of the laboratory assessments at each sentinel sites, questions were organised into 25 distinct indicators where the score of each indicator was determined by taking the average score of all questions grouped with that specific indicator. These indicators were then further grouped into 5 broad dimensions for analysis (1). Quality Management (2). Financial and Human Resources (3). Data and information management (4). Microbiology Testing Performance (5). Surveillance Capacity. The score of each dimension is calculated as the average of all indicator scores grouped within the dimension. The structure of each dimension and their associated indicators is described in Table 2. For the microbiology testing performance dimensions, only tests that are relevant to identifying the seven bacteria under surveillance are assessed: *Staphylococcus aureus, Streptococcus pneumoniae, Klebsiella pneumoniae, Salmonella* spp.*, Acinetobacter* spp.*, Escherichia coli* and *Burkholderia pseudomallei*. The full list of questions grouped under each indicator, full formulas for calculating indicator and dimension scores and the complete LAT used in this study can be found in the Supplementary files.

**Table 1:** Code tree and descriptions.

Themes and sub-themes	Description
Microbiology supplies and logistics	
Limited access to microbiology supplies	*Inability to obtain adequate supplies due to cost, lack of availability or supply chain issues*
Bulk procurement	*Purchasing supplies in large quantities to reduce the cost per unit*
Challenges with reagents or consumables utilisations	*Difficulties in conducting tests due to supply stockouts or short expiration dates*
Low demand for microbiology services	*Small microbiology test volumes*
Financial sustainability of clinical microbiology	
Operational cost	*Cost of ongoing activities in a microbiology laboratory*
Funding sources	*Financial resources contributing to AMR surveillance system operations*
External support dependency	*Reliance on resources provided by private organisations to aid microbiology laboratories reduces sustainability of quality service*
Data representativeness and bias	
Surveillance design	*Criteria or methods to systematically collect, analyse, interpret and report cases of AMR*
Healthcare workers’ behaviours	*Healthcare workers’ prescribing practice, diagnostic testing practice, and adherence to guidelines*
Data utilisations	*Using aggregated AMR surveillance data to inform decision-making at the national or sub-national level*
Data representation	*The extent in which the collected data can indicate the true level of AMR in the population targeted by the surveillance system*
Communications and engagements	
Laboratory staff-clinician interface	*The interactions between the laboratory staffs and clinical staffs*
Patient-focus	*Prioritisation of patient management and treatment*
Microbiology service utilisations	*The rate or frequency of requesting diagnostic microbiology tests*
Microbiology workforce and performance	
Staff competency	*Microbiology staffs have adequate qualifications and skills to fulfill their roles*
Microbiology guidance	*Training to enhance microbiology service*
Laboratory staff sufficiency	*Adequate number of laboratory staffs to provide quality microbiology service*

**Table 2:** Dimensions and indicators evaluated by the laboratory assessment tool.

**1. Quality Management**
1.1 Document control procedures
1.2 Quality procedures
1.3 Equipment management procedures
1.4 Reagent management procedures
1.5 Accreditations achieved
1.6 Audits performance
**2. Financial and Human Resources**
2.1 Available budget for routine lab and surveillance functions
2.2 Staff training and supervision resources
2.3 Staff qualifications adequacy
2.4 Staff sufficiency
**3. Data and information management**
3.1 Types of data collected and reported
3.2 Data analysis capacity
3.3 Laboratory information management system capacity
**4. Microbiology Performance**
4.1 Specimen collection and handling procedures
4.2 Blood culture procedures and competency
4.3 Cerebrospinal fluid procedures and competency
4.4 Bacterial identification testing procedures and competency
4.5 Antibiotic susceptibility testing procedures and competency
4.6 Minimum inhibitory concentration tests procedures and competency
4.7 Internal quality control procedures and performance
4.8 External quality assurance procedures and performance
**5. Surveillance capacity**
5.1 Reporting and notification capacity
5.2 Specimen shipping and transportation capacity
5.3 Surveillance network participation
5.4 Outbreak response capacity

#### Ethical considerations

Ethical approval for the research protocol was obtained from the National Ethics Committee for Health Research in Cambodia (NECHR No. 311). All informants were provided an information sheet, informed about the objective of the study, and were asked to review and sign a consent form prior to the interviews. Consent was also obtained for audio-recording when permitted. Informants kept a copy of both the information sheet and consent form for participation in the study. Identifying information was removed to ensure anonymity and confidentiality as indicated by the informants. To provide additional confidentiality, informants were provided with the option of not being quoted. Confidentiality was maintained throughout the whole process of data collection, analysis and publication or report.

## Results

### Key informant’s perspectives

#### Characteristics of key informants

In total, 25 individuals (18 HMs and 7 SPs) participated in the qualitative study and took part in the KIIs. Some KI fulfilled dual roles as both AMR focal points and as laboratory staffs (laboratory technicians or managers); the full list of KI and their associated background is outlined in Table 3. The response rate to the respondent validation survey was 68% (17/25).

**Table 3:** Key informants’ characteristics.

Type of informants	Number of KI interviewed (*N* = 25)
High-level management (*N* = 18)	
Head of laboratory	4
Head of microbiology laboratory	3
Chair or vice-chair of hospital AMR committee	4
Chair or vice-chair of hospital IPC committee	3
Government official at Communicable Disease Control Department	3
NGO staff	1
Surveillance personnels (*N* = 7)	
AMR focal point	4
Data analyst	1
Laboratory technician	2
Laboratory manager	3
Clinical microbiologist and physician	1

#### Microbiology supplies and logistics

Accessing quality laboratory supplies were widely agreed to be a constraint for the surveillance system. Although Cambodia has improved its access to growth media with the development of the central media making laboratory (CMML), obtaining certain microbiology reagents or consumables remain a burden for sentinel sites due to their high cost (16). In Cambodia, public healthcare facilities have two primary means of procuring medical supplies: by direct purchasing from private distributors, and through a benefit package offered by the government through the central medical store (CMS).

Despite the addition of microbiology materials in the essential medicines list in 2018, key informants explained that microbiology supplies were rarely procured by the central government as haematology and biochemistry laboratory supplies were cheaper and perceived to be of higher priority. Therefore, sentinel sites are reliant on supplies purchased from private distributors who often sell these items at a high price for profit. The main factors attributed to driving the cost of microbiology supplies is a combination of weak supply chain, small number of microbiology laboratories and inability to make bulk purchases. Consumables and reagents that need to be imported such as antibiotic disks are particularly susceptible to an increase in price due to cost of shipping through cold storage, distance and import tax. Additionally, informants reported that the small number of tests being conducted, an incremental financing system, and short expiration dates of internationally imported supplies hindered their ability to make bulk purchases; thus, private distributors can charge a premium for these items. The COVID-19 pandemic further exacerbated this issue as it severely disrupted the global supply chain. As a result, majority of informants perceived inadequate access to microbiology supplies as the biggest burden for the AMR surveillance system.

“But if we order from abroad it takes 1–2 months. And another problem, we are unable to order in smaller quantity either. They would not deliver it unless, we either add extra money because of tax registration or cost of import. Or for another choice, we have to order in their required volume. Which is a problem because we do not need that much, and they might be expired if we just store them” – SP.

To address this problem, some sentinel sites have participated in supply sharing with other microbiology laboratories to prevent stockouts. Some informants perceived that the burden of microbiology supply is a systematic issue requiring a top-down approach to solve. It was suggested that centralisation of microbiology supply procurement and policy-level changes could reduce the cost of importing microbiology supplies. It is believed that these strategies would enable better access to microbiology supplies across all means of procurement.

“One solution we try, is that we usually borrow from other labs, for example one box of this reagent or that reagent. One place we have borrowed from recently is [redacted] lab. This allows us to solve the problem for when we just need small amounts of material and without having to order a lot in advance” – HM.

#### Financial sustainability of clinical microbiology service

The quality and output of the national AMR surveillance system in Cambodia requires well-functioning microbiology laboratories as the surveillance data are directly transferred from routine microbiology data at each sentinel site. Informants expressed that one of the barriers to conducting AMR surveillance is the poor financing of microbiology services in the public health sector. Seven out of the eight sentinel sites participating in the national AMR surveillance system are part of the public healthcare system where healthcare is funded by three main sources (1). Government general revenues (2). External aid (3). Individual out of pocket payments (OOP) for receiving services. The informants indicated concerns with the current model as revenues made from OOP, the predominant source of finance for healthcare, is not able to cover the cost of materials needed to perform tests. This resulted in several KI who advocated for increasing the price of microbiology tests being done at public hospitals. However, they believed that there is an administrative barrier as approval at the level of provincial health departments is required.

“Another main source is from user fees, however, the amount of money we received back from the hospital is not enough to cover the cost of operation especially not to buy supplies for running tests for AMR. In addition to this, I am planning to raise the price for microbiology tests” – HM.

Many informants indicated the importance of partnerships with international organisations and external donors to alleviate the cost of supplies. However, this creates a dependency on external funding which may affect the quality of the surveillance system in the long term. Informants particularly highlighted concerns about the discontinuation of the Diagnostic Microbiology Development Program (DMDP). Since 2008, DMDP has played a pivotal role in building the microbiology capacity of Cambodian public healthcare facilities by helping set up diagnostic microbiology in public hospitals, donating laboratory supplies, and providing technical guidance and mentorships to five sentinel sites; in some cases, DMDP also provided direct financial assistance. Informants reported that the quality of data produced by the surveillance is expected to decline due to the termination of DMDP’s support, as these sentinel sites have relied on the resources provided by DMDP.

“Ever since DMDP stopped supporting us, we experienced a big issue with finding funds to operate our microbiology lab” – HM.

“I think that some sentinel sites will start to be more frugal about their use of supply, for example, if this one kit is used for one sample, they might split for 2. Another is the reduction of QC (quality control). So, I think quality will go down. Now, it is still new, the absence of DMDP. The quality might not go down much. However, in the long term, I believe that the quality will go down a lot because of the absence of DMDP” – HM.

#### AMR surveillance data representativeness and bias

There was broad consensus among participants in the HM group that the data produced from the current AMR surveillance system is unable to represent the burden of AMR of each sentinel site’s catchment population. Participants indicated three different factors which bias the data captured by the surveillance system. First, participants believe that current passive laboratory-based surveillance would result in too many undetected cases and that a syndromic or case-based surveillance is more appropriate for AMR cases. In addition, the surveillance system only captures AMR cases in the inpatient departments. Last, participants were concerned about physicians’ behaviours on requesting microbiology tests. It was noted that many physicians would request for a microbiology test only when their patients did not recover at initial antibiotic therapy despite meeting the designated criteria for doing a microbiology test. Although this is the recommended practice in some international clinical guidelines, initial antibiotic therapy can drastically reduce the sensitivity of microbiology tests when detecting pathogens. As a result, some senior officials expressed their hesitancy in utilising the data for decision-making at the national and regional level. Furthermore, one KI also stated that a hospital-based surveillance system would not be able to collect nationally-representative AMR data as community AMR cases are not captured by these sentinel sites.

“If we use the data to represent, I think it is still biased. In order for them to request culture, then patient must be very ill or sick. Doctors will treat them first and if they do not get better, then they will request for culture. Therefore, organisms with low resistance will be treated and is not captured. So only the high resistant pathogens get picked up” – HM.

However, one senior staff acknowledged that, while the current surveillance data may not accurately represent the burden of AMR in the hospital’s population, the sentinel site would also lack the resources to process an increase in samples if the surveillance system were to expand the design to encompass a larger population for testing or to implement diagnostic stewardship programs to increase data representation.

“I think that there is already a lot of obstacles in terms of financial resources with testing the amount of IPD samples we received. If we were to culture OPD samples as well, then the lab would need to double the resources in order to implement this change” – HM.

#### Communications and engagement

This theme emerged from the respondent’s views on how the surveillance system could be improved. Due to the multi-disciplinary nature of AMR surveillance, a strong relationship is required between clinicians and laboratory staff. Participants advocated for a strong and consistent engagement between these two groups to increase utilisation of microbiology service for patient management of AMR cases. A knowledge gap exists for clinicians and laboratory staff since Cambodian physicians, during their pre-service education, are not typically trained in clinical microbiology and its significance. Conversely, laboratory staff would also need to be aware of the clinical practice guidelines that clinicians utilise. Eliminating the knowledge gap were stated to be very important both by laboratory staff and physicians in the study. Sharing of preliminary results such as Gram stain results to the responsible clinicians through instant messaging and establishing an immediate notification system for critical results were also reported as essential for promoting a stronger relationship between the teams. Overall, it was suggested that consistent and timely communications across these two teams can create trust and increase the perceived values of microbiology, resulting in better cooperation and higher sample volumes. This ultimately leads to more data being sampled by the surveillance system, thus increasing its accuracy in monitoring AMR.

“I think clinical engagement, patient-focus engagement is really important. How can we help clinicians? Regular feedback and discussions. […]. The lab is culpable too; they often do not fully engage with the needs of the clinician” – HM.

The importance of laboratory staff-to-clinician interface is demonstrated by participants’ perspectives on “Lab round” activities. These are activities that are implemented in 5 sentinel sites specifically to promote the interactions between clinicians and microbiology teams. Physicians are invited to visit the microbiology laboratory and facilitate knowledge exchange with the goal of improving patient outcome. Although many participants acknowledged the benefits of lab rounds, it was reported that COVID-19 had halted this activity and has not yet been resumed.

“But back when there was a group that was made for this activity, this lab round would happen every day. Even when there is not a positive sample, they would come, and we can discuss what the doctors might want to know about lab process or pathogen that the doctors might question. Because in the lab, we have data about disease history connected to a lab case, so if the patient has been here a year ago, we will know what lab case they had. So, this is useful for the doctors” – SP.

#### Microbiology workforce and performance

One of the biggest challenges for many AMR surveillance systems in LMICs is the limited capacity in diagnostic microbiology. In Cambodia, participants perceived the identification of non-fermenting Gram-negative bacilli and serotyping of *Salmonella* spp. to be the biggest challenge. On top of this, microbiology staff reported that more training opportunities on bacterial identification techniques which are cost-effective or inexpensive are essential for the sustainability of AMR surveillance.

“There should be training on new techniques, ones that we are not doing. I think the kind of new techniques that we can use with our current amount resources would be great” – SP.

Informants broadly agreed that the AMR surveillance system lacks a body of experts to provide technical guidance for microbiology testing at laboratories. This is a critical issue for the surveillance system since clinical microbiology is a constantly evolving field with frequent updates to guidelines and methodologies. Currently, the NRL provides reference function such as quality control and assurance. The role of providing training and guidance on bacterial identification and antimicrobial susceptibility testing (AST), originally fulfilled by DMDP, is now a gap in the surveillance system due to the DMDP’s exit. Laboratory staff who participated in our study were concerned about the absence of guidance for these techniques as sentinel sites do not have the capacity to independently find training resources and update their protocols according to international standards. One participant indicated that the current AMR surveillance system is also missing a microbiology expert for governance. It was reported that the system needed a senior microbiologist to manage the system alongside epidemiologists due to the complex nature of microbiology data. In summary, it was perceived that the AMR surveillance system needs more technical guidance for microbiology and data analysis.

“First, I want the AMR surveillance system to have a real expert on AMR and microbiology. We have a lot of laboratory technicians, but we do not have anyone who actually has a lot of experience and is an expert in microbiology. Additionally, we can analyse the data but whether the analysis or result is correct, we do not know” – HM.

#### Laboratory capacity assessment of sentinel sites

A laboratory-based AMR surveillance system’s performance is highly dependent on the capacity of laboratories to accurately diagnose pathogens and characterise their resistance to antibiotics. Therefore, microbiology laboratories at participating sentinel sites were assessed on their capacity to provide quality microbiology service and conduct AMR surveillance. Six out of the eight sentinel sites were assessed for this study. Five sentinel sites were microbiology laboratories at provincial hospitals under the public healthcare system while one laboratory was a microbiology laboratory at a non-governmental paediatric hospital.

**Figure 2:**
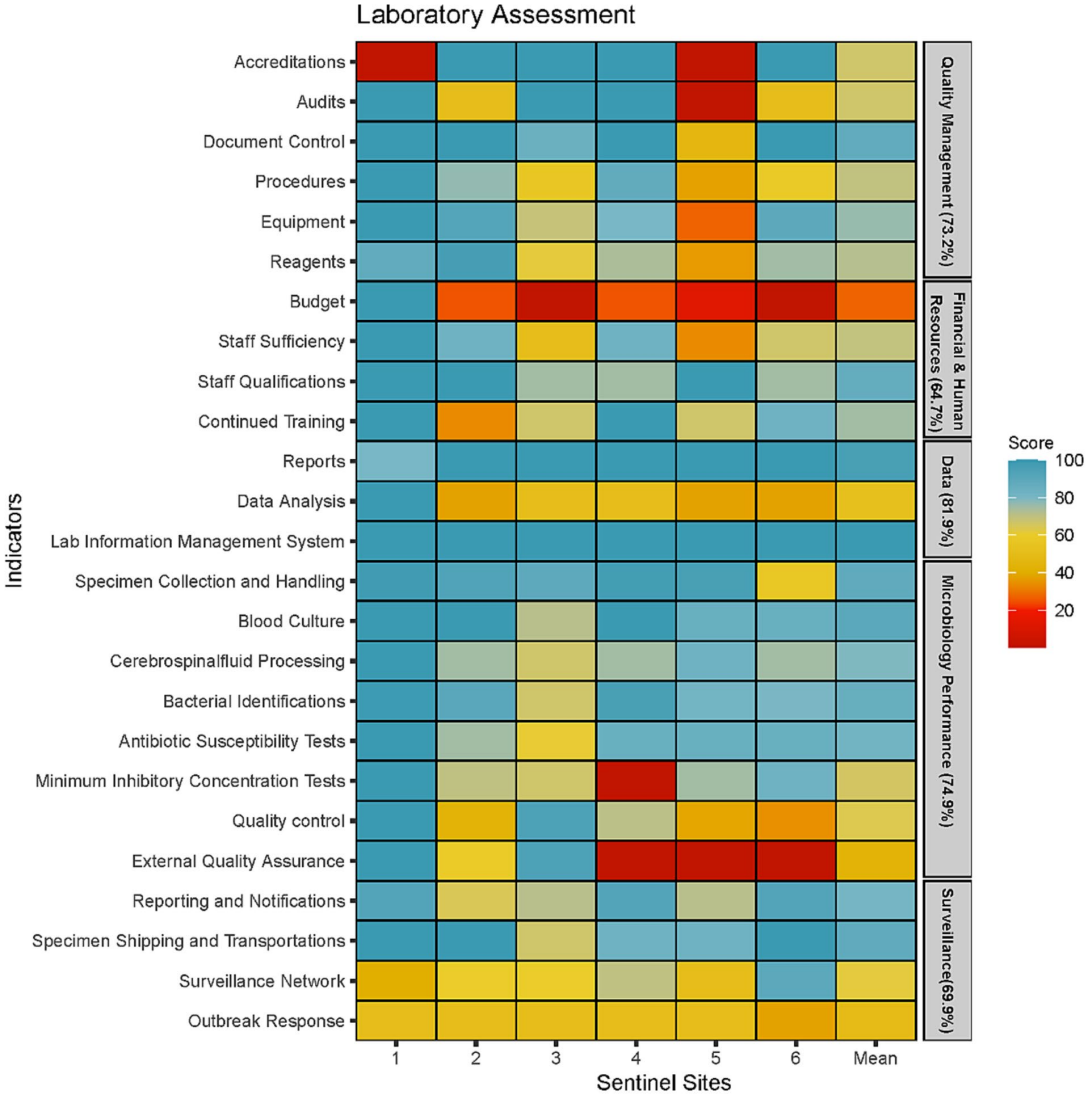
Microbiology laboratory capacity assessment at six sentinel sites represented by five areas of laboratory operations (Quality Management, Financial and Human Resources, Data Management, Microbiology Testing Performance and Surveillance Capacity).

The weakest performing area was the availability of budget and funding with an average score of 27% (Figure 2). Only one sentinel site had a separate budget allocated for AMR surveillance. Four laboratories did not have adequate budget for equipment maintenance; only a few or none of their equipment are regularly serviced. One of the core functions of the surveillance system is to refer isolates to the NRL for quality control and storage in a biobank. However, this function has been halted since 2020 due to lack of funds reflecting the poor financing of microbiology services in Cambodia. Another indicator that was also affected by the poor financing was the EQA indicator as only three laboratories could afford to enrol in EQA programs.

Two indicators that scored particularly low was the surveillance network and outbreak response indicator. Five laboratories did not have guidelines for responding to cases of AMR outbreaks. Instead, each outbreak is managed on a case-by-case basis, often without standardised response. Furthermore, a public health laboratory network had not been set up and, therefore, there was no framework for systematically referring isolates from critical pathogens for public health purposes. Data analysis was another indicator which scored less than 60% in five sentinel sites. While all five laboratories were able to produce antibiograms and describe AMR trends, they did not frequently produce this report to communicate with clinicians or infection prevention control (IPC) teams. Furthermore, the ability to conduct descriptive statistics were limited to a small number of laboratory staff who received additional training or education. Last, the use of AMR surveillance data to inform facility level empirical antimicrobial therapy was non-existent in five out of the six sentinel sites.

The assessment showed that microbiology performance was particularly well-developed in five laboratories with a score of 75% or more in all indicators except for Minimal Inhibitory Concentrations (MIC), EQA and quality control. All six laboratories had appropriate SOPs and equipment to identify the seven priority organisms to the genus level and conduct AST using antibiotic disks. We found that the microbiology staff possess good competencies with the basics of microbiology tests such as bacterial identifications through morphological and biochemical tests, and AST using disk diffusion techniques. Difficulties around identification was only encountered when isolates could not by identified with the standard biochemical test. The main reasons provided for the low scores for MIC tests were inadequate or expired reagents to perform these procedures, rather than the lack of staff competency. However, it was observed that sentinel sites only had the capacity to use E-strips to measure MICs while the ability to perform microdilutions was lacking in five out of the six sentinel sites. Inadequate supplies also resulted in 5 sentinel sites foregoing quality control procedures such as using control strains to test the viability of media and testing reagents. This resulted in low scores for the quality control indicator. In addition, five laboratories did not have a stock management system monitoring supply levels of reagents and other consumables and forecasting the volume and time to procure them. The absence of such system could play a role in weakening the microbiology supply chain as suppliers could use this information to maintain adequate inventory.

## Discussion

Previous studies have suggested that a major obstacle to AMR surveillance in low-resource settings is the limited access to equipment, consumables and reagents adapted to tropical conditions (14, 17). In our study, adaptation of microbiology supplies was not identified as a significant barrier to supply access or availability. This may be attributable to the fact that microbiology laboratories are situated exclusively in urban areas with sufficient cold chain storage network. Our study found that access to microbiology supplies is more limited by a weak supply chain and high cost of procurements. Low demands of microbiology supplies drive costs, reducing access by poorly funded laboratories. Although Cambodia’s essential medicines list, which contains both priority medicines and laboratory supplies, also includes diagnostic microbiology supplies, lack of perceived importance of microbiology further limits support by government benefit packages and programs. This overall lack of access to microbiology supplies can lead to laboratories resorting to unstandardised utilisation practices which compromises the quality of surveillance outputs (18). While accessing imported microbiology supplies is still a barrier to AMR surveillance in Cambodia, the in-country production of culture media at the CMML has played an essential role in facilitating the AMR surveillance system by expanding access to essential microbiology supplies in Cambodia (16). More initiatives focusing on local access to microbiology supplies like the CMML should be implemented to further strengthen Cambodia’s AMR surveillance system.

The study revealed that clinical microbiology at public healthcare facilities is not a sustainable service due to a combination of weak financing, dependence on external funding and low microbiology service utilisations. Therefore, the quality and consistency of data being captured by the system is severely reduced when external funding is absent, or supply costs increase. These are both scenarios which our study have revealed to occur frequently. A study analysing Southeast Asian hospitals showed that the cost of conducting identification and AST on a single specimen ranged between $22 and $31 (19). This is costly relative to the cost of antibiotic treatments in LMICs further discouraging microbiology utilisation both from the patient’s and hospital’s perspectives (20). Along with a limited access to microbiology supplies, this results in a constant cycle of low supply and low demand which is detrimental to the quality of poorly financed clinical microbiology services. In the short term, incorporating diagnostic stewardship to increase demand and implementing a more robust inventory and stock management system will help alleviate some of the financial burden faced by microbiology. In the long term, developing a sustainable business model of clinical microbiology at public healthcare facilities will ensure a more effective laboratory-based AMR surveillance system that adequately captures AMR data over long periods of time. Future studies looking to address this issue should aim to investigate the financing and cost–benefit analysis of microbiology services in public healthcare facilities in order to evaluate the most cost-effective strategies for conducting microbiology in limited resourced settings but without causing financial hardship on people who need the service. Additionally, the microbiology diagnostic market in Cambodia should be studied to ensure a healthy market with predictable supplies and demands. Lastly, implementing strategies to take advantage of economies of scale may contribute to solving these sustainability and financing issues encountered by clinical microbiology in the public sector.

Our study found that stakeholders were not keen on using data captured by the surveillance system for AMR-related programs and policies due to its perceived inability to accurately represent the national burden of AMR. Diagnostic stewardships will also help alleviate this problem as it increases the number of samples captured by each sentinel sites. The number and representativeness of the sentinel sites (e.g., adding rural sites) could also be addressed. Countries such as Thailand and Australia deploy multiple types of surveillance system to target specific population groups on top of their laboratory-based surveillance with the goal of creating a nationally representative database (21, 22). These are strategies which the national AMR surveillance system in Cambodia could model after. However, sufficient resources will be needed to implement such robust surveillance system.

Results obtained from the laboratory assessment showed the negative effects of the barriers identified in the qualitative component of this study. Specifically, quality control and assurance measures were reduced to save supplies for clinical diagnostics. Data analysis of aggregated AMR data was also found to be a gap and will need to be improved at both national and sub-national level across Cambodia. Capacity building for data analysis may also motivate sentinel sites to collect higher quality data collection since it would allow for the sentinel site to extract more value from AMR data beyond storage and fulfilling a duty for the surveillance system. In contrast to other LMICs, Cambodia’s national AMR surveillance system is not limited by a lack of competency to conduct microbiology tests (14, 23). We noted that the microbiology staff are well-qualified and have extensive experience with the basics of microbiology tests. Limitations are more on the number of staff and qualified microbiologists, access to appropriate reagents and supplies when it comes to bacterial identification and AST.

**Table 4:** Summary of barriers, facilitators and recommendations identified by framework analysis.

**Barriers**
Poor supply chain systems and a small demand for microbiology limits access to microbiology supplies
An unsustainable financing system means that the quality of data collected varies with the amount of fund available to the laboratories collecting data
Infrequent utilisations of diagnostic microbiology by clinicians and a passive surveillance system that only collects inpatient data limits the surveillance from being representative of hospital population
Absence of local microbiology experts to provide continuous training and guidance on microbiology techniques and data interpretations
**Facilitators**
Quality and consistent interactions between clinicians and laboratory staffs are important for increasing microbiology utilisations and the number of samples captured by the surveillance system
**Recommendations**
Procuring microbiology supplies that need to be imported from abroad should be centralized to a third-party institution to reduce cost and ensure consistent availability to all microbiology laboratories in Cambodia
Invest more resources into implementing lab rounds at hospitals to promote collaborations between clinical and laboratory staffs
Invest resources to implement active surveillance in the form of case-based or syndromic based surveillance at sentinel sites to enhance data representation
Implement cheaper or inexpensive microbiology techniques for bacterial identifications or antibiotic susceptibility tests and avoid building capacity for new techniques that require expensive equipment or maintenance

Our study aligns with previous studies on the importance of promoting consistent and practical engagement between clinicians and laboratory staff (24, 25). A strong laboratory staff-clinician relationship enables a more effective AMR surveillance by increasing proper utilisation of clinical microbiology services. When microbiology teams are more focused on the analytical stage rather than as members of the team concerned with clinical management of a patient, a negative relationship may form between the two teams. This may result in clinicians perceiving microbiology as expensive and an obstacle for patient treatment. In contrast, training on clinical microbiology and the appropriate use of microbiology testing for clinicians and other healthcare workers could also facilitate stronger cooperations between the laboratory staff and clinicians, promote the importance of microbiology testing, and increase the number of samples being captured by a laboratory-based surveillance system. However, while strategies for increasing sampling rate is important for the surveillance system, Cambodia’s AMR surveillance system also suffers from a small workforce since the number of laboratory staff trained in microbiology is limited. As such, interventions that increase sampling and microbiology utilisation will need to be accompanied by strategies that address the workforce issue such as employing automated equipment or increasing opportunities for education on microbiology.

The barriers and facilitators identified in this study further reinforced many of the core building blocks of an AMR surveillance system which have been identified during Georgia’s experience in setting up their AMR surveillance system (Table 4) (25). By demonstrating the cost-savings associated with appropriate utilisation of diagnostic microbiology, Georgia was able to increase the budget allocated for microbiology at public hospitals; this is a strategy which can also be effective for tackling the financing issues faced by Cambodia’s microbiology laboratories. Additionally, Georgia has had success in developing a sustainable and stable supply chain for their microbiology services by centralising their procurement process. The success observed in Georgia suggests that similar recommendations identified in this study could yield positive results for Cambodia as well. One of the limiting factors identified in this study is the lack of informants’ experience in analysing and utilising of AMR surveillance data. AMR surveillance data have not been used by any health institutions in Cambodia to influence policies and interventions before the time of this study. As such, it is unlikely that the present participants have enough experience to give a comprehensive perspective on using data produced by the AMR surveillance system. Consequently, this also limits the identification of gaps in the data being produced by the surveillance system. Additionally, majority of KI participating in the study were staff involved with clinical microbiology or public health surveillance. Clinician- or physician-informants also had additional roles within IPC or AMR committees. These participants are therefore well-aware of hospital AMR surveillance and its importance. Their perspectives may not be representative of many other clinicians at the sentinel site such as nurses or physicians who are not directly involved in IPC or AMR.

## Conclusion

AMR surveillance is one of the five strategic objectives in the WHO’s Global Action Plan on AMR as it plays a critical role in generating data for evidence-based decision-making to combat AMR. It is therefore important for countries to address barriers that prevent an effective implementation of a national AMR surveillance system. This study adds new evidence for health officials in Cambodia to utilise when developing an improvement strategy for the surveillance system. Access to supplies imported from abroad needs to be facilitated by reducing its cost to enable quality-assured microbiological testing. This may be addressed by applying economies of scale, for instance, centralising the procurement process of microbiology essentials through a third-party institution or the national reference laboratory as have been demonstrated by other LMIC’s surveillance systems (26). The cost of operating a quality microbiology service is too high for public healthcare facilities in Cambodia suggesting a need to develop a more financially sustainable model for microbiology services in limited resourced settings, but ideally without passing the burden to its people. Data generated by the surveillance system are not representative of the national population hindering its use to develop antimicrobial treatment guidelines and monitoring the effectiveness of ongoing antimicrobial stewardship programs. Diagnostic stewardship and expanding surveillance to capture cases beyond inpatient departments at referral hospitals are both viable strategies to increase representativeness but must be accompanied with adequate resources to support such development. These findings can serve as a source of inspiration for other countries with limited resources and facing challenges with AMR surveillance, helping them to identify relevant and actionable barriers and facilitators for their own contexts.

### Scope statement

This is the first study to provide a comprehensive understanding of the key factors influencing the national AMR surveillance system in Cambodia. To implement a successful AMR surveillance system in low resourced settings, a thorough analysis of each factor is necessary. These findings will benefit all stakeholders in shaping AMR detection and surveillance in Cambodia. Furthermore, this study is directly related to strengthening a country’s process to generate evidence-based public health policies and programs as outlined in the scope of the Infectious Disease section of the journal. The assessment tools and its methodologies used in this study can be used by other countries interested in evaluating its microbiology laboratories or their laboratory-based surveillance system.

## Data availability statement

The raw data supporting the conclusions of this article will be made available by the authors, without undue reservation.

## Ethics statement

The studies involving humans were approved by National Ethics Committee for Health Research. The studies were conducted in accordance with the local legislation and institutional requirements. The participants provided their written informed consent to participate in this study.

## Author contributions

SM: Conceptualization, Data curation, Formal analysis, Methodology, Project administration, Resources, Visualization, Writing – original draft, Writing – review & editing. CS: Data curation, Formal analysis, Validation, Writing – review & editing. SL: Data curation, Formal analysis, Validation, Writing – review & editing. JJ: Writing – review & editing. GK: Conceptualization, Writing – review & editing. DC: Conceptualization, Methodology, Resources, Supervision, Writing – review & editing. CC: Conceptualization, Funding acquisition, Investigation, Methodology, Project administration, Resources, Supervision, Writing – review & editing. PI: Conceptualization, Funding acquisition, Investigation, Methodology, Project administration, Resources, Supervision, Writing – review & editing.

## Glossary

AMR: Antimicrobial Resistance
AST: Antibiotic Susceptibility
CMML: Central Media Making Laboratory
EQA: External Quality Assurance
HM: High-Level Management
IPC: Infection Prevention and Control
LAT: Laboratory Assessment Tool
LIMS: Laboratory Information Management System
LMIC: Low- and Middle-Income Countries
MIC: Minimum Inhibitory Concentrations
NRL: National Reference Laboratory
OOP: Out-of-Pocket
QC: Quality Control
SOP: Standard Operating Procedure
SP: Surveillance Personnels
WHO: World Health Organisation
