# Supplementary material for: The barriers and facilitators of implementing a national laboratory-based AMR surveillance system in Cambodia: key informants’ perspectives and assessments of microbiology laboratories
Source: Front Public Health. 2023 Dec 21;11:1332423. doi: 10.3389/fpubh.2023.1332423 (PMC10764616; doi:10.3389/fpubh.2023.1332423)
Supplement: Supplementary file 2 [file Table_2.DOCX]

Questionnaire for Key Informant Interviews at Sentinel Sites

These questions should be used as a guide to conduct interviews for key informants at sentinel sites such as the head of laboratory, vice-head of laboratory, AMR staffs and infection and prevention control committee’s member or any other staffs who may be involved with AMR surveillance at the sentinel site. Depending on the needs and relevance, the interviewer may use all the questions in this questionnaire or just certain questions for the key informant interviews.

| Date of Interview |  |
| --- | --- |
| Interviewer ID |  |
| Province |  |
| Sentinel site |  |
| Position of the participant |  |

1. Could you give a brief introduction about background and your role within the AMR surveillance system?

____________________________________________________________________________________________________________________________________________________________________________________________

1. Could you describe your sentinel site’s role in the AMR surveillance system?

________________________________________________________________________________________________________________________________________________________________________________________________

1. In your opinion, do you think there is adequate staff to conduct AMR surveillance at your lab?

Yes

No

If not adequate, in what way?

_______________________________________________________________________________________________________________________________________________________________________________________

1. Does the lab have training or programmes for its staffs?
   1. Is this adequate? What do else do you think they should be trained in?

| Types of training | Frequency (0 if doesn’t have) |
| --- | --- |
| General professional development |  |
| External development (Conference, workshop and external education) |  |
| Internal development (on-site training and journal club) |  |

Financial Resources

1. Does the lab have a separate budget allocated for AMR surveillance?

Yes

No

_______________________________________________________________________________________________________________________________________________________________________________________

1. In your opinion, is there enough budget to adequately conduct AMR surveillance at the lab?

Yes

No

Consumables and Reagent purchase:_________________________________

Equipment and Maintenance:_______________________________________ Human resource (staff education or labour):____________________________ Other:_________________________________________________________

1. Describe the sources of your financial support for AMR surveillance.

_______________________________________________________________________________________________________________________________________________________________________________________

1. For AMR surveillance in general, do you receive any support from external institutions or organizations? How do they support AMR surveillance at your sentinel site?

_______________________________________________________________________________________________________________________________________________________________________________________

Supply Management

1. What kind of problems and challenges, if any, does your lab face when it comes to supply management, equipment and reagents or consumables?
   1. How do you solve these problems?
   2. How do you think these problems could be avoided in the future?

_______________________________________________________________________________________________________________________________________________________________________________________

AMR surveillance data

1. For microbiological testing of AMR, how does the lab validate its results? Do you think that this current method of validation is adequate to provide quality data?

_______________________________________________________________________________________________________________________________________________________________________________________

1. Do you think that doing confirmation testing useful? What else would help to boost quality assurance for AMR surveillance at your laboratory?

_______________________________________________________________________________________________________________________________________________________________________________________

1. What kind of output or report is being produced from the AMR surveillance data? Where is it sent to and how often?

_______________________________________________________________________________________________________________________________________________________________________________________

1. Do you think that the current AMR surveillance data is representative of the surveillance target population?

_______________________________________________________________________________________________________________________________________________________________________________________

General Summary and Perception of AMR surveillance

1. In your opinion, how would you rate the success of AMR surveillance at your laboratory from 1 to 10? Do you have any recommendations for improving the AMR surveillance system?

_________________________________________________________________________________________________________________________________________________________________________________

1. How useful is the AMR surveillance is for the hospital and its communities?

_________________________________________________________________________________________________________________________________________________________________________________
