# Supplementary material for: The barriers and facilitators of implementing a national laboratory-based AMR surveillance system in Cambodia: key informants’ perspectives and assessments of microbiology laboratories
Source: Front Public Health. 2023 Dec 21;11:1332423. doi: 10.3389/fpubh.2023.1332423 (PMC10764616; doi:10.3389/fpubh.2023.1332423)
Supplement: Supplementary file 3 [file Table_3.DOCX]

Supplementary files: Distributions of questions making up indicators and formulas for computing score of each indicator.

| **1. Quality Management** |
| --- |
| 1.1 Document control procedures |
| *Questions: Q2.1-2.9* |
| 1.2 Quality procedures |
| *Questions: Q2.10-2.45* |
| 1.3 Equipment management procedures |
| *Questions: Q6.1-Q6.29* |
| 1.4 Reagent management procedures |
| *Questions: Q5.1-Q5.34* |
| 1.5 Accreditations achieved |
| *Questions: Q1.37* |
| 1.6 Audits performance |
| *Questions: Q1.28, Q1.29, Q1.31, Q1.33-Q1.35* |
| **2. Financial and Human Resources** |
| 2.1 Available budget for routine lab and surveillance functions |
| *Questions: Q1.18-Q1.27* |
| 2.2 Staff training and supervision resources |
| *Questions: Q9.29-9.32* |
| 2.3 Staff qualifications adequacy |
| *Questions: Q9.17-Q9.19, Q9.21* |
| 2.4 Staff sufficiency |
| *Questions: Q9.1-Q9.9, Q9.20* |
| **3. Data and information management** |
| 3.1 Types of data collected and reported |
| *Questions: Q4.1-Q.4.22* |
| 3.2 Data analysis capacity |
| *Questions: Q4.23-Q4.25, Q4.39* |
| 3.3 Laboratory information management system capacity |
| *Questions: Q4.31-Q4.38* |
| **4. Microbiology Performance** |
| 4.1 Specimen collection and handling procedures |
| *Questions: Q3.1- Q3.39* |
| 4.2 Blood culture procedures and competency |
| *Questions: Q7.1* |
| 4.3 Cerebrospinal fluid procedures and competency |
| *Questions: Q7.2* |
| 4.4 Bacterial identification procedures and competency |
| *Questions: Q7.5-Q7.17, Q7.36* |
| 4.5 Antibiotic susceptibility tests procedures and competency |
| *Questions: Q7.31-Q7.32* |
| 4.6 Minimum inhibitory concentration tests procedures and competency |
| *Questions: Q7.33* |
| 4.7 Internal quality control procedures and performance |
| *Questions: Q7.1-Q7.17, Q7.31-Q7.33* |
| 4.8 External quality assurance procedures and performance |
| *Questions: Q7.1-Q7.17, Q7.31-Q7.33* |
| **5. Surveillance capacity** |
| 5.1 Reporting and notification capacity |
| *Question Q10.16-Q10.22* |
| 5.2 Specimen shipping and transportation capacity |
| *Question: Q3.40-Q3.46* |
| 5.3 Surveillance network participation |
| *Questions: Q10.2, Q10.4, Q10.6, Q10.11* |
| 5.4 Outbreak response capacity |
| *Question Q10.8, Q10.10, Q10.12-Q10.15* |

Table: Distribution of questions making up each indicator and dimension. Note: Question is indicated by the question number given to each question in the LAT form. In regards to the microbiology testing performance dimension, due to the structure of the questionnaire, each question may be associated with multiple response. We decided to split the scoring system into measuring testing capacity and quality control performance. Therefore, for indicator 4.2-4.6, scores are calculated for questions that are answered in the following categories (as indicated by the LAT): Staff, SOPs, Equipment, Reagents/Test kits. For indicator 4.7 only response to the category called Quality control are used to compute the score, while the indicator 4.8, responses in the category named ‘External Quality Assessment’ are used to compute scores.

Example calculation of scores

| **Indicator name: Data analysis capacity** | | |
| --- | --- | --- |
| **Question** | **Response** | **Assigned score** |
| *Q4.23* | 1 | 100% |
| *Q4.24* | 3 | 0% |
| *Q4.25* | 2 | 50% |
| *Q4.39* | 3 | 0% |
| Calculation: (100+0+50+0) ÷ 4 = 37.5% | | |
| **Data analysis capacity score: 37.5%** | | |

| **Dimension name: Data management** | |
| --- | --- |
| **Indicators** | **Score** |
| Types of data collected and reported | 100% |
| Data analysis capacity | 37.5% |
| Laboratory information management capacity | 50% |
| Calculations: (100+37.5+50) ÷ 3= 62.5 | |
| **Data management score: 62.5%** | |
